# DEEP SCOPE: A Framework for Safe Healthcare Design

**DOI:** 10.3390/ijerph18157780

**Published:** 2021-07-22

**Authors:** Ellen Taylor, Sue Hignett

**Affiliations:** 1 The Center for Health Design, Concord, CA 94520, USA; 2 Design School, Loughborough University, Leicestershire LE11 3TU, UK; S.M.Hignett@lboro.ac.uk

**Keywords:** patient safety, facility design, falls, HF/E design principles, theoretical framework

## Abstract

Thinking in patient safety has evolved over time from more simplistic accident causation models to more robust frameworks of work system design. Throughout this evolution, less consideration has been given to the role of the built environment in supporting safety. The aim of this paper is to theoretically explore how we think about harm as a systems problem by mitigating the risk of adverse events through proactive healthcare facility design. We review the evolution of thinking in safety as a safety science. Using falls as a case study topic, we use a previously published model (SCOPE: Safety as Complexity of the Organization, People, and Environment) to develop an expanded framework. The resulting theoretical model and matrix, DEEP SCOPE (DEsigning with Ergonomic Principles), provide a way to synthesize design interventions into a systems-based model for healthcare facility design using human factors/ergonomics (HF/E) design principles. The DEEP SCOPE matrix is proposed to highlight the design of safe healthcare facilities as an ergonomic problem of design that fits the environment to the user by understanding built environments that support the “human” factor.

## 1. Introduction

Twenty years after the seminal Institute of Medicine (IOM) reports “To Err is Human” and “Crossing the Quality Chasm,” there continue to be questions about safety and the speed of progress (or lack thereof). According to some, we have investigated preventable adverse healthcare events to make sense of the factors that contribute to error [1], but in too many evaluations of patient safety, the search for causation ends with a blame-and-retrain mentality [2]. In fact, a key message in the IOM studies was emphasizing error as a systems problem and identifying human factors as an important component of patient safety [3]. Two decades later, there continue to be challenges in using human factors/ergonomics (HF/E) principles and understanding the latent conditions that underlie the safety problems that need to be solved. Designing a safe healthcare facility is no exception.

### 1.1. The Challenges of Complexity

Safety scientists have documented the movement from behavioral and linear Newtonian thinking (representative of a complicated system) into newer views of complexity [4]. While the terms are sometimes used interchangeably, the difference between complicated and complex systems is important. Complicated systems are described as stable, closed to the environment, knowable, and controllable with a pre-existing order of any outcome, whereas complex systems are more than a sum of the parts—always changing due to relationships and interactions between parts [4,5,6]. However, in past efforts to improve safety, we have often searched for empirical research that supported an improved outcome for a specific intervention. By focusing on this limited view of complex systems (whether work systems, productions systems, or other), there may be a danger of missing the larger multi-factorial problems that exist.

Further, our understanding of the complexity in systems (such as the delivery of care) may be exacerbated by organizational silos, often departments or service lines that operate independently, avoid sharing information, and do not always recognize their potential role in upstream or downstream events. An incomplete understanding of a situation or problem may be equally prevalent in healthcare design, where both user input and technical expertise may be siloed. While not specific to facility design, the following sentiment may resonate with the healthcare design community:

*Although healthcare providers work together, they are trained in separate disciplines, where the primary emphasis is the mastery of the skills and knowledge to diagnose ailments and render care. In the pursuit of becoming as knowledgeable and skillful as possible in their individual disciplines, a challenge facing nursing, medicine, and the other care specialties is to be aware of the reality that they are but one component of a very intricate and fragmented web of interacting subsystems of care where no single person or entity is in charge*.

[7] (p. 3)

In a real-world context of limited time and financial pressures, it becomes tempting to focus on simple fixes—the low-hanging fruit—rather than address the fundamental underlying issues that take a more prolonged period to study [8]. Even the best-intentioned architects and designers may seek evidence-based solutions only to end up asking about the elusive black and white answer to solve a problem.

### 1.2. An Evolution in Conceptualizing Patient Safety

In the past three decades, there has been an evolution in how we think about patient safety in healthcare, even though “patient safety” was first included as a MeSH (Medical Subject Heading) term in 2012 [9]. Whereas the field of human factors/ergonomics has largely influenced safety in other industries (e.g., nuclear power, aviation), its use in the complex arena of healthcare is more recent.

One description of the evolution of systems approaches for patient safety [10] traced thinking back to Reason’s 1990 accident causation model [11], Vincent, Taylor-Adams, and Stanhope’s 1998 framework for analyzing risk and safety in medicine [12], the use of the Haddon matrix at the turn of the century [13], and the subsequent development of the Systems Engineering Initiative for Patient Safety (SEIPS) model of work system design for patient safety in 2006 [10].

#### 1.2.1. Work System Design: Human Factors/Ergonomics for Safety in Healthcare

The SEIPS model was originally developed as a result of the lack of models to guide studies to empirically examine work system design [10], and the model has continued to evolve to better incorporate patient activity [14] and the care processes across the patient journey [15]. SEIPS is based on the Donabedian structure–process–outcome framework [16,17], and the model categorizes the work system, process, and outcomes and includes technology and tools, tasks, the organization, the person, and the environment. The SEIPS model references the layout of the environment (e.g., visibility), noise, lighting, temperature, humidity and air quality, and workstation design, and proposes that plans are reviewed for workflow and questions are asked about the physical environment sources that promote error or safety. The original model promoted the structure of the work system, building on prior research for balanced job design to reduce stress [18,19]. It was described for application both proactively and reactively by focusing on the design of work and has subsequently been revised to more clearly focus on patient work [14,15].

#### 1.2.2. Resilience and Safety-II

There has also been work [20] to track an evolution from “old” thinking about human error to “new” thinking in healthcare-based resilience engineering [21], in which the focus is not just on what went wrong (Safety-I) but better understanding the everyday performance that usually succeeds (Safety-II). Safety-II considers the ability of systems to adapt to variation, disruption, and degradation of expected conditions [22,23]. One can see the transition through papers about accident barrier classification and analysis [24], to a recognition that a reactive approach was insufficient, necessitating accident prevention and a proactive approach [25], to safety as a dynamic non-event (i.e., the absence of events) using a framework of resilience. Importantly, the reactive approach of Safety-I should be complemented (not replaced) by proactive Safety-II approaches that attempt to develop ways to support things that “go right” [21]. With calls for the recognition of patient safety science as a profession [26], recent advances include practical activities of a safety professional through the Safety-II lens [27].

From a resilience perspective, the built structure is one part of a functioning system, such that a hospital needs to adapt through continual rebuilding—both organizationally and physically [28]. Even so, the role of structures is not often described in Safety-II, and according to Hassler and Kohler [29], “*the composition and dynamic of the built environment prove to be very complex and attempts at description remain very general*”. Proponents have urged taking into account that those remote from the clinical frontline base solutions on “work as imagined”, rather than “work as done” [21], and there continues to be criticism about conceptualizing safety events in healthcare as a linear chain of events rather than drawing on a larger body of safety science [30].

## 2. Theoretical Underpinning

A safety risk assessment (SRA) is a process that has been developed to proactively consider the influence of healthcare facility design in mitigating the risk of harm to users (e.g., patients, staff) [31,32], yet challenges remain. As previously described, safety is a systems problem. This section presents the theoretical underpinning of an expanded model for mitigating the risk of adverse events, furthering a HF/E systems approach. Based on earlier work, falls continue to be used as the “case study” example.

### 2.1. Designing to Mitigate Risk

Previous publications describe the development and testing of a safety risk assessment for healthcare facility design to proactively consider six safety issues (i.e., falls, patient handling, infection prevention, medication safety, harm associated with behavioral health, and security) that may be influenced by the underlying conditions of the built environment: [31,33]. While testing the “Falls” module of the SRA toolkit, there were often difficulties in solving design problems [33]. For example, during hypothetical scenarios and pilot tests, participant discussions included bathroom location (proximity versus identification), floor transitions (shower curbs or smooth transitions), and existing standards. These were often tradeoff decisions, and supporting research was inadequate to address the “lived-in” challenges raised by participants evaluating the tool.

Because bathroom location influences the structural grid and overall unit size, a primary discussion for inpatient unit design was room layout: inboard (hallway side), outboard (window side), or nested toilets (between rooms on hallway and window side), as well as whether the bathroom is located on the headwall or footwall [34]. One participant commented that there was an intuitive response to locate the bathroom as close as possible to the patient, without definitive research to support the decision. Visibility into the bathroom may result in a loss of patient privacy, and, in many projects, the desire for an identifiable bathroom was sacrificed for proximity and privacy.

Multiple views were also offered about bathroom/shower floor transitions and real-world implications (e.g., wiping water from the floor). In the case of one pilot site, nursing leadership stated that clinical procedures would guide the decision of a curbless shower and nurses would wipe the floor, yet there was not a follow-on discussion of what design features would best support nurses in this task. The reverse was the case in the hypothetical scenario test. Introducing a curb to eliminate the wet floors was not discussed with respect to facilitating patient movement into the shower.

Grab bars or emergency pull cords may be placed according to code or manufacturer recommendations for accessibility, but, without awareness of the physical limitations of movement or manipulation that might be experienced by an ill or aging patient, these may not be suitably located. Participants referenced this solely as a code issue. These examples illustrate that when framed as an environmental condition, the interaction of end-users in the system is often lost. Introducing HF/E design principles is proposed to reframe the conversation into a problem of design that fits the environment to the user [35].

### 2.2. The SCOPE Model

As presented in a systematic mixed studies review for patient falls [36], safety can be conceptualized in a SCOPE model—the complexity of interactions with the organization, people, and environment (Safety = Complexity × (Organization + People + Environment)). The SCOPE model expanded Hignett’s [37] Dial-F systems model describing building design and stability at the core of mitigating the risk of falls. People (the primary “human” factor in design) possess an interrelated set of intrinsic conditions that both influence and are influenced by the built environment. The SCOPE framework was divided into three broad categories of organization (i.e., policies and procedures), people (i.e., patients, staff, caregivers), or environment (the physical setting in which activities take place) [36]. As the term environment can have different meanings, four subset “components” were used in the SCOPE framework: the workspace envelope as the wider workplace including the building characteristics, adjacencies, and space constraints; personal workspaces that include the layout of the staff or patient “workstation” or immediate area of use; products, such as the selection/specification of equipment, furniture, or controls; and the ambient environment—thermal, air, noise, and illumination considerations. While the categories of organization, people, and environment have the potential to address a systems approach, they are potentially discrete units [38] that may benefit from additional integration. This paper reframes the SCOPE framework for safety more definitively as an ergonomic design problem, continuing to use falls as an example.

### 2.3. HF/E Design Principles

To understand fit, it is important to understand the active participants (patients and staff). Designing for an unknown future user in a HC facility is complex and must consider the general conditions of human performance, behavior, and user characteristics. Five HF/E healthcare design principles have been adapted from pre-SEIPS work [39] to re-establish a foundation of HF/E design principles to address user fit. These principles, originally proposed for manufacturing [40] and office environments [41,42], include:

- Optimizing opportunities for movement;
- Minimizing manipulation time;
- Minimizing the need for human strength;
- Minimizing perception time, and
- Minimizing decision-making time.

#### 2.3.1. Optimizing Opportunity for Movement (Mv)

The human body is not built to stay in the same position for lengthy periods and optimal design must balance the need for movement in patient care duties and sitting or standing for charting or other stationary activities [39,41,42]. Equipment and materials should be conveniently located and easily accessible, with technology (e.g., cell phones, laptops) allowing for freedom of movement from workstations [39,41,42]. In some instances, the speed of movement needs to be considered. According to Sanders and McCormick [43], response time is considered as a combination of reaction time (i.e., from signal onset to the beginning response) and movement time (i.e., the beginning response through to the completion of the response). This aspect of movement can also be related to decision-making. Accordingly, designers should:

- support healthy/neutral postures that provide comfort without annoyance, allowing flexibility in furniture (e.g., chairs, standing workstations, resilient flooring) [39,41,42,44];
- place all things that a user must operate with their hands in front of the user, at elbow height, and within reach [39,41,42,44];
- locate visual displays within a normal line of sight and cone of easy eye rotation [45].

#### 2.3.2. Minimizing Manipulation Time (Ma)

Manipulation includes physical affordances and constraints to optimize use. Wickens [44] posits that structural (static) and functional (dynamic) anthropometric data can help designers prevent awkward positions (i.e., heights, reach, grip, clearances) while recognizing human variability (i.e., age, gender, ethnicity, occupation). However, dimensional characteristics (e.g., reach) do not guarantee the ability to lift or manipulate an object, and mechanical forces also must be taken into consideration [44].

With respect to design, parts or equipment should be easy to move, easy to grip/grasp, and should not tangle, while materials should not be weak, easy to bend (unless intended), or likely to chip or crack [39,40,41]. In addition, the transfer of training (e.g., how to use equipment) should be considered so that previously acquired skills can be applied to new products or workstation layout to avoid confusion and loss of efficiency [39,40]. Identified options [44] for design consideration include:

- designing for the extreme (e.g., clearance for the largest, reach for the smallest);
- designing for adjustability (e.g., seats);
- designing for the average (e.g., a registration counter);
- designing for a percentile (e.g., the 5th or 95th to define upper and lower limits).

#### 2.3.3. Minimizing the Need for Human Strength (St)

Strength is influenced by motivation and will [45], and is most often associated with muscles in the arm, leg, or back and can be dynamic (e.g., lifting) or static (e.g., holding, gripping) [43]. A lack of strength can result in musculoskeletal injury or whole-body fatigue [44]. Biomechanical analysis is one approach for assessing dynamic capacity for infrequent manual handling tasks, while physiological approaches are often used for frequent tasks done over a period of time [43]. Psychophysical approaches take into account biomechanical and physiological stresses but also consider perceived stress [43,45]. Studies have shown that strength exhibits an accelerated decline starting at age 51–55 (an 80% decrease from peak strength), with a 60% strength capacity (as compared to peaks) by ages 71–75 [43]. This has implications for both patients and an aging healthcare workforce. As a result, designers should incorporate mechanical devices to reduce or eliminate the need for human strength [39,41,42].

#### 2.3.4. Minimizing Perception Time (Pe)

Information is collected by the senses (a bottom-up process of what is there through visual legibility, audibility, familiar representations) and is influenced by expectations that are a result of short- and long-term memory, a top-down process of what should be there through discriminating features, context, and redundancy [44,46]. Research in this area [39,40] suggests that designers should:

- Understand that hidden or invisible parts are sometimes forgotten (e.g., small fonts on display monitors);
- Use visual discrimination such as size or color coding to form families of parts that belong together and enhance stimulus response for reduced reaction time (e.g., red for alarms);
- Recognize that touch (texture and size) can be a discriminating factor (e.g., sanded door knob finish to indicate no entry).

#### 2.3.5. Minimizing Decision-Making Time (Dm)

Decision-making is influenced by mental effort and attentiveness: selective, focused, and divided [44,47]. Researchers have described the decision-making task as choosing from more than one alternative through information available relative to the options, and choice may be associated with uncertainty and no clear best option [44]. Decision-making follows the delivery of perceptual information, which is interpreted through the working memory (impacted by capacity and time) [44,47]. While decision-making in situ varies from decision theory and choice behavior in controlled settings, some cognitive task analysis methods, inclusive of those individuals performing the work, have been developed to bridge this gap (e.g., field observations, work domain analysis, goal-directed task analysis, critical decision methods) [48]. As mental models help organize the execution of a task, and task visibility is important in creating a mental model, researchers [39,40] suggests that designers should:

- Consider the user’s mental model and recognize that diverse tasks result in different mental models to achieve different things with differing priorities (e.g., visibility, different alarm sounds);
- Minimize the number of (or co-locate) components and related tools (also saving space) to reduce choice reaction time (e.g., code button at the bed);
- Locate work elements in sequential order with task items that belong together in close physical proximity (e.g., crash carts) to improve spatial compatibility and improve stimulus response;
- Incorporate visual, tactile, or auditory feedback to indicate that the task was completed (e.g., electronic sound for touchscreen functions).

## 3. Results: An Expanded Model for Safety

The addition of these design principles results in an expanded framework for safety: DEEP SCOPE (DEsigning with Ergonomic Principles).

### 3.1. The Development of DEEP SCOPE

The DEEP SCOPE model is proposed by integrating the relationships of the organization, people, and environment previously set forth in the SCOPE model for falls [36]. The expanded thinking provides a way to synthesize findings for safety into a systems-based model for building. By better understanding building design as a systems problem, architects and designers can be better positioned to define the problem to address appropriate fit for an evidence-based and human-centered design.

Numerous interventions were categorized according to layers of stability across the categories of organization, people, and environment in the original SCOPE model for falls. These design conditions are now further categorized according to the principles of ergonomic design. For example, the principle of movement related to falls would include walking surfaces (floor materials and transitions, weather/contamination protection), tripping hazards (clutter, cords, equipment), understanding organizational policies for surface maintenance (cleaning, repair, accessibility of supplies), recognizing necessary movement aids for people (walking aids, bedside commodes), and facilitating the reach of personal items. Transfer assistance to prevent falls could be categorized as manipulation (“manipulating” people as compared to inanimate objects), and this would include providing necessary space to support organizational policies of patient handling, along with wide doors to allow assisted ambulation (which could also be movement, highlighting that not every intervention exists as a one-to-one relationship). Other considerations would include the manipulation of objects: call systems, doors (while the patient is attached to an IV or using a walking aid), and grab bars placed within a suitable reach. Strength, in the context of the SCOPE model for falls, would include the room/bathroom configuration, toilet location in the bathroom, the use of grab bars to support weaker patients (also in manipulation for reachability), and the use of patent lifts to aid both patients (ambulation) and staff (at risk of falling from reflex reactions during assistance). Organizational policies for mobilization programs (and where this takes place) could influence design decisions (e.g., activities on unit hallways or in patient rooms, versus an occupational/physiotherapy area).

As it pertains to mitigating the risk of falls, perception in the expanded model would include fall alert visual cues inside and outside the patient room, the ability to leave doors open, lighting, decisions for technology to reduce noise (e.g., alarms, paging), and a recognition of patient conditions of care (e.g., medications) that result in an overestimation of abilities or other changes in perception. Lastly, the model suggests that decision-making to mitigate the risk of falls would most likely include considerations in an organizational context. From a design perspective, this would necessitate an awareness of organizational policies that may result in spatial or other design considerations. For example, the use of patient sitters and facilitation of family presence may require space for furniture; video monitoring may require space for monitors at a centralized location, as well as the necessary infrastructure for technology; fall documentation may require space at the bedside and/or another location; universal versus customized protocols may require storage space; segregation of populations and intent for patient placement may influence unit size and configuration, and access to patient education materials may need to be considered in the context of technology, whiteboards, or placement of other written materials. Design also should take into account unit layout and surveillance options as needed, especially as they pertain to the general workflow of care (fitting the environment to the user). Researchers found that the physical environment was one of four work process constraints contributing to the risk of falls as a result of workarounds that included written and mental chunking schemas, bed alarms, informal querying of the previous care nurse, and informal video and audio surveillance [49]. Such workarounds have been called “*first order problem solving that adapts work to cope with inefficiencies”* [50] (p. 140).

#### 3.1.1. The DEEP SCOPE Model

**Figure 1:**
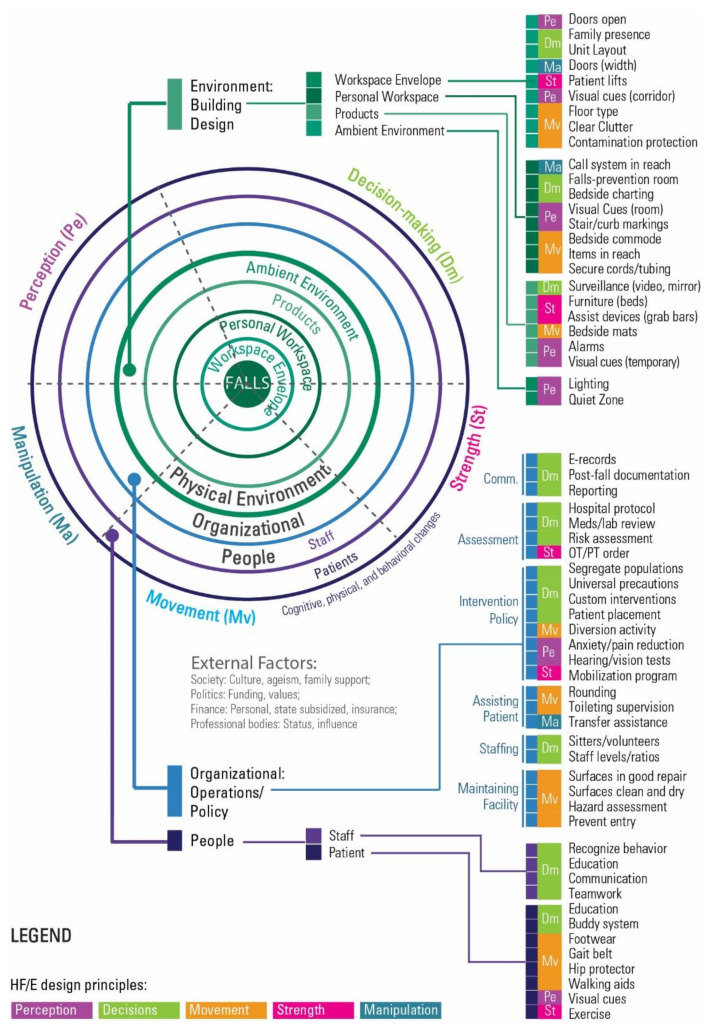
DEEP SCOPE: Designing with Ergonomic principles—Expansion of the SCOPE model (Safety = Complexity * (Organization + People + Environment)) [36].

The resulting DEEP SCOPE model (Figure 1) builds on the SCOPE systems model by incorporating the HF/E design principles. The updated model adds a color-coded design principle that supplements the three categories of organization, people, and environment.

**Figure 2:**
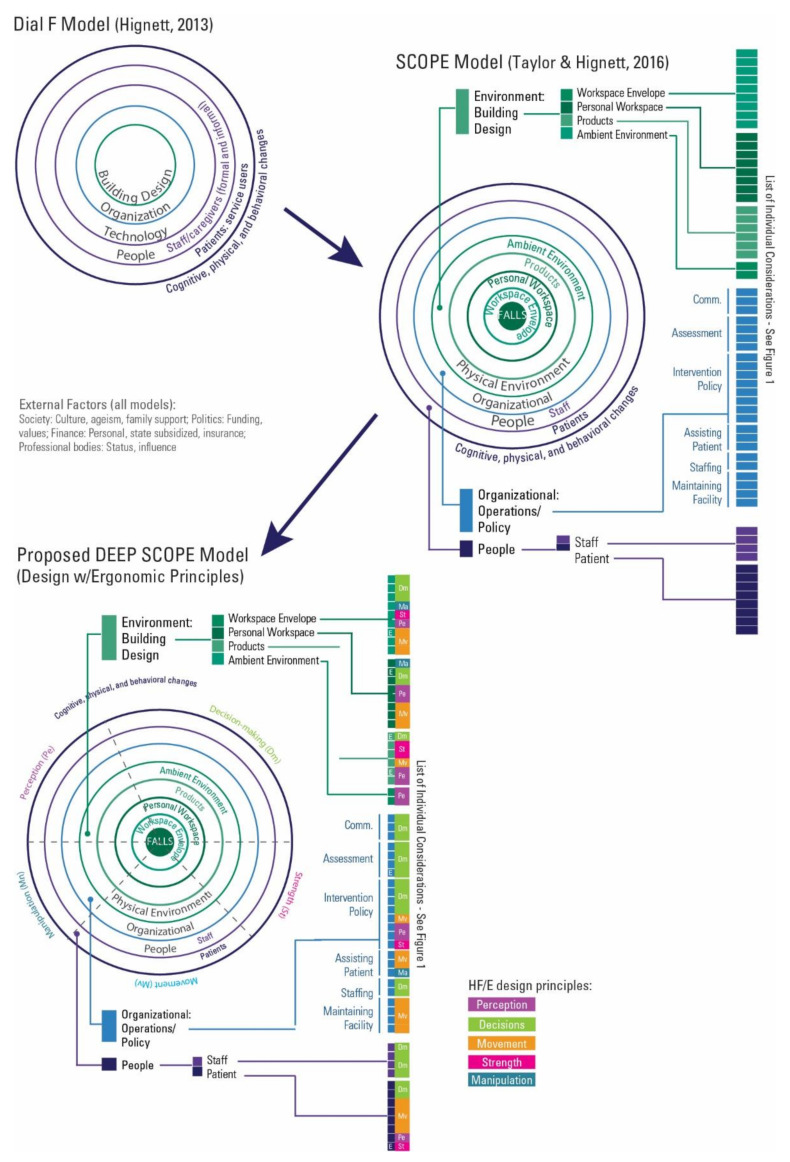
The evolution of the SCOPE and DEEP SCOPE model.

As shown, there is a range of interventions that cross all of the HF/E design principles, as well as the subcomponents of the physical environment. The organizational considerations are marked by a prevalence of decision-making interventions, whether associated with communication, culture, patient assessment, or patient-based interventions. People-based interventions focus primarily on the patient and span a range of the HF/E design principles. Figure 2 illustrates the evolution of the SCOPE framework.

#### 3.1.2. The DEEP SCOPE Matrix

**Figure 3:**
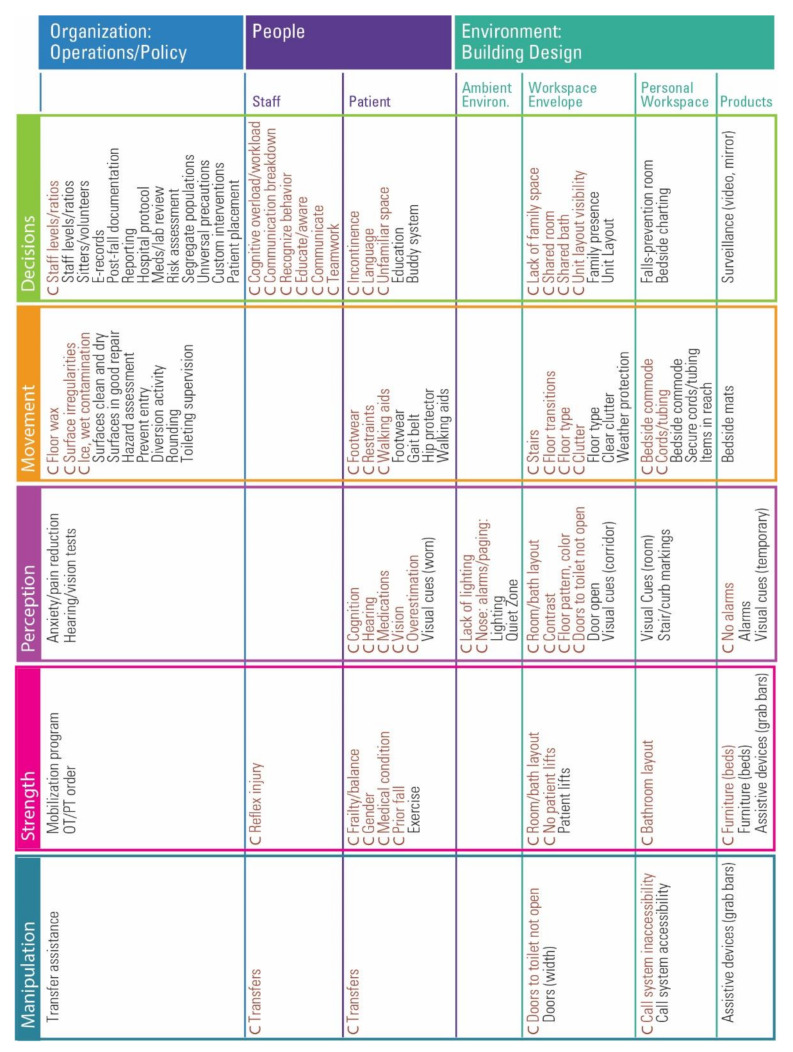
The DEEP SCOPE Matrix for Falls. (C: Correlated for falls).

A second visualization takes the form of a matrix (Figure 3), furthering the framework for design considerations. The DEEP SCOPE matrix includes the correlates of falls and suggests the alignment of interventions that have been tested or used as part of a multifactorial bundle. It allows for interventions to be placed with more than one principle (e.g., grab bars support weak patients *and* are placed within reach.)

## 4. Discussion

The understanding of falls is complex. The aim of this paper was to theoretically explore how we think about the challenge of safety in healthcare facility design rather than to experimentally quantify the effect of specific interventions. When design teams are assembled, HF/E experts are typically not considered as a necessary or value-adding member. Further, experts in HF/E may not be well versed in the process for designing built environments (although the lack of experience in designing a building project is not restricted to the discipline of HF/E). The proposed DEEP SCOPE model presents the opportunity to frame safe building design as an HF/E problem, not just an architectural problem of structure, space, and articulated function. The model offers a way to incorporate design principles used in HF/E with evidence-based design strategies. The blended approach can be used by the team as part of a systems approach to mitigating risk. In this paper, the model has been populated using the case study example from the previously published SCOPE model. As a bridge integrating HF/E and evidence-based design, the DEEP SCOPE model advances a systems-based process, purposefully developed to address the design of the built environment, to more fully address the complexity of safety and falls in healthcare. As a result, it is important to frame this approach within the context of prior models.

### 4.1. The Proposed Model in Context

The literature surrounding thinking for patient safety has evolved since the development of Reason’s [11] accident causation model, often used as a basis for the role of the environment as a barrier to errors. However, while this model recognizes system influences, Reason posits a sequential approach that originates in imperfect decisions and line management deficiencies, further hampered by preconditions and unsafe acts that pass through a limited window of accident opportunity [11].

The strength of sequential thinking for accident causation is the etiology of accidents and adverse events with descriptions of contributing factors, while the lack of discussion of processes and guidance for system redesign is a weakness [10]. As a result, guidance for system redesign was addressed through the SEIPS models [10,14], where the work systems (including the internal and external environment) influence processes that subsequently influence outcomes. While the benefit of the SEIPS is the focus on the system design and description and the resulting effect on processes and outcomes, its authors stated that its weaknesses included its framework—a descriptive model with no specific guidance as to the critical elements [10]. The original SEIPS model referenced the use of plans and questions to determine the contribution of the environment to patient safety; many papers that have cited the model as a framework in the study design have offered little detail on the influence of the built environment in their results.

### 4.2. Designing Safe Facilities—An HF/E Problem

Several recent studies have incorporated the SEIPS model to frame how the built environment supports safe and effective workflow as part of a complex system [51,52,53,54], but the framework of the most recent SEIPS model maintains its focus on work systems, albeit temporal and across settings. The physical environment is still shown in the SEIPS 3.0 model, but it seems to become lost across the outlined journey. However, the design of the environment may take on an even more important role, as the design of each healthcare workplace becomes the stage for each process [55]. In contrast, Hignett’s Dial-F model proposes that safety includes layers of permanence, with the patient being the most transient and the built environment being most stable [37]. The permanence of the built environment results from the significant financial investment required to renovate or build new facilities. A design that tries to fit the user to the environment, rather than designing the environment to support the use, can thus continue to negatively influence safety over time [37]. Dial-F makes a leap to highlight the prominent role of the environment, but was not intended as a tool for healthcare facility design.

While the evolution of safety models offers significant contributions in delivering healthcare, and each model is logical in the context of an intended audience, the intended audience has never included architects or designers. As a result, the built environment is too often considered the existing condition. With few avenues for recourse, users develop workarounds, fitting themselves to the environment. What is missing from these prior approaches is both a hierarchy of decision-making for interactions with the environment (which we have addressed through HF/E design principles) and guidance for proactive design (the conditions and built environment interventions to be considered during an evidence-based design process for healthcare facilities). The DEEP SCOPE theoretical framework furthers our understanding of safety, establishing building design as an HF/E problem by:

- establishing the stability of the built environment and identifying HF/E environment categories (workplace envelope, personal workspace, products, and ambient environment);
- categorizing design interventions into three interacting categories (organization, people, and environment);
- creating connections across the organization, people, and environment through five HF/E design principles (manipulation, strength, movement, perception, decision-making).

### 4.3. Strengths, Limitations, and Future Research

As discussed throughout the paper, there is no shortage of safety models, but no model has been developed to address the specific concerns of designing built environments for the safe delivery of care. This model may challenge design teams to think differently, and may require additional expertise on the team, but the DEEP SCOPE matrix creates a novel approach and visual roadmap for teams to consider established HF/E design principles alongside evidence-based organizational strategies, strategies that may facilitate tasks and activities for staff and patients, as well as built environment strategies across a range of permanence within a facility. The theoretical framework is an integrated approach for proactively considering the opportunities for safer building design.

There are limitations with the proposed model, as the case study example only addresses patient falls. As previously discussed, the Safety Risk Assessment process for healthcare settings considers other areas of safety, as well. There is an opportunity to develop the evidence-based strategies within this model to include issues such as infection prevention, medication safety, and others. The benefit of continuing the development of the framework for specific topics is that the approach allows the integration of HF/E design principles across multiple issues, again advancing a systems approach for designing safe healthcare facilities.

Additionally, this paper only presents the theoretical approach and does not present an example fusing a specific facility design project. However, another paper incorporates the theoretical framework and DEEP SCOPE matrix for the analysis of data collected as part of a project to proactively design a patient room for stability as a fall-reduction strategy [56]. The DEEP SCOPE framework will continue to be used as the room design develops, and this may serve as an example of how teams can use the matrix to inform design decisions in projects, whether addressing a specific room type (e.g., a medication prep room) or an overall unit layout (e.g., designing a medical–surgical unit to mitigate risk).

## 5. Conclusions

The DEEP SCOPE model is intended to proactively advance safer healthcare facility design. There is significant worth in discussing evidence-based design in healthcare facilities as a HF/E problem. This goes beyond “work as imagined” (which is often what is provided in the project brief) and offers opportunities to address “work as done”—what may promote or impede desired behaviors for safety, rather than trying to modify behavior (or environments) after the fact. The proposed model offers a framework that has been purposively developed as a proactive approach for safety in facility design that serves as a bridge for the domains of EBD and HF/E. In summary, an understanding of HF/E conditions through the SCOPE and DEEP SCOPE models can advance a framework for more fully considering the ongoing problems that we face with improving safety by understanding built environment solutions that support the “human” factor.

## Data Availability

Not applicable.

## References

[1] Henriksen K., Kaplan H. (2003). Hindsight Bias, Outcome Knowledge and Adaptive Learning. Qual. Saf. Health Care.

[2] Catchpole K. (2013). Spreading Human Factors Expertise in Healthcare: Untangling the Knots in People and Systems. BMJ Qual. Saf..

[3] Carayon P. (2011). Handbook of Human Factors and Ergonomics in Health Care and Patient Safety.

[4] Dekker S. (2011). Patient Safety: A Human Factors Approach.

[5] Dekker S. (2014). Safety Differently: Human Factors for a New Era.

[6] Simon H.A. (1962). The Architecture of Complexity. Proc. Am. Philos. Soc..

[7] Henriksen K., Dayton E., Keyes M.A., Carayon P., Hughes R.G., Hughes R.G. (2008). Understanding Adverse Events: A Human Factors Framework. Patient Safety and Quality: An Evidence-Based Handbook for Nurses.

[8] Henriksen K. (2011). Opportunities and Challenges in the Pursuit of Patient Safety. Handbook of Human Factors and Ergonomics in Health Care and Patient Safety.

[9] Willis S.R. (2012). NLM Technical Bulletin.

[10] Carayon P., Hundt A.S., Karsh B.-T., Gurses A.P., Alvarado C.J., Smith M., Brennan P.F. (2006). Work System Design for Patient Safety: The SEIPS Model. Qual. Saf. Health Care.

[11] Reason J. (1990). The Contribution of Latent Human Failures to the Breakdown of Complex Systems. Philos. Trans. R. Soc. B Biol. Sci..

[12] Vincent C., Taylor-Adams S., Stanhope N. (1998). Framework for Analysing Risk and Safety in Clinical Medicine. BMJ.

[13] Brasel K.J., Layde P.M., Hargarten S. (2000). Evaluation of Error in Medicine Application of a Public Health Model. Acad. Emerg. Med..

[14] Holden R.J., Carayon P., Gurses A.P., Hoonakker P., Hundt A.S., Ozok A.A., Rivera-Rodriguez A.J. (2013). SEIPS 2.0: A Human Factors Framework for Studying and Improving the Work of Healthcare Professionals and Patients. Ergonomics.

[15] Carayon P., Wooldridge A., Hoonakker P., Hundt A.S., Kelly M.M. (2020). SEIPS 3.0: Human-Centered Design of the Patient Journey for Patient Safety. Appl. Erg..

[16] Donabedian A. (1966). Evaluating the Quality of Medical Care. Milbank Mem. Fund Q..

[17] Donabedian A. (1988). The Quality of Care: How Can It Be Assessed?. JAMA.

[18] Carayon P., Smith M.J. (2000). Work Organization and Ergonomics. Appl. Ergon..

[19] Smith M.J., Sainfort P.C. (1989). A Balance Theory of Job Design for Stress Reduction. Int. J. Ind. Ergon..

[20] Carthey J. (2013). Understanding Safety in Healthcare: The System Evolution, Erosion and Enhancement Model. J. Public Health Res..

[21] Braithwaite J., Wears R.L., Hollnagel E. (2015). Resilient Health Care: Turning Patient Safety on Its Head. Int. J. Qual. Health Care.

[22] Hollnagel E., Woods D.D. (2006). Epilogue: Resilience engineering precepts. Resilience Engineering–Concepts and Precepts.

[23] Woods D.D., Hollnagel E. (2006). Prologue: Resilience engineering concepts. Resilience Engineering. Concepts and Precepts.

[24] Hollnagel E. Accidents and Barriers. Proceedings of the Seventh European Conference on Cognitive Science Approaches to Process Control.

[25] Hollnagel E. (2004). Barriers and Accident Prevention.

[26] Smith K.M., Valenta A.L. (2018). Safety I to Safety II: A Paradigm Shift or More Work as Imagined?. Int. J. Health Policy Manag..

[27] Provan D.J., Woods D.D., Dekker S.W.A., Rae A.J. (2020). Safety II Professionals: How Resilience Engineering Can Transform Safety Practice. Reliab. Eng. Syst. Saf..

[28] Hollnagel E. (2014). Resilience Engineering and the Built Environment. Build. Res. Inf..

[29] Hassler U., Kohler N. (2014). Resilience in the Built Environment. Build. Res. Inf..

[30] Wiig S., Braithwaite J., Clay-Williams R. (2020). It’s Time to Step It up. Why Safety Investigations in Healthcare Should Look More to Safety Science. Int. J. Qual. Health Care.

[31] Taylor E., Joseph A., Quan X., Duffy V. (2012). Design for Patient Safety—Considering a Patient Safety Risk Assessment. Advances in Human Aspects of Healthcare.

[32] The Center for Health Design Online Safety Risk Assessment Toolkit|A Process to Mitigate Risk. https://www.healthdesign.org/sra.

[33] Taylor E., Quan X., Joseph A. (2015). Testing a Tool to Support Safety in Healthcare Facility Design. Procedia Manuf..

[34] Pati D., Harvey T.E., Reyers E., Evans J., Waggener L., Serrano M., Saucier R., Nagle T. (2009). A Multidimensional Framework for Assessing Patient Room Configurations. HERD Health Environ. Res. Des. J..

[35] Hignett S., Carayon P., Buckle P., Catchpole K. (2013). State of Science: Human Factors and Ergonomics in Healthcare. Ergonomics.

[36] Taylor E., Hignett S. (2016). The SCOPE of Hospital Falls: A Systematic Mixed Studies Review. HERD Health Environ. Res. Des. J..

[37] Hignett S. (2013). Why Design Starts with People. Health Found..

[38] McNeese M.D., Zaff B.S., Citera M., Brown C.E., Whitaker R. (1995). AKADAM: Eliciting User Knowledge to Support Participatory Ergonomics. Int. J. Ind. Ergon..

[39] Carayon P., Alvarado C., Hundt A.S. (2003). Reducing Workload and Increasing Patient Safety through Work and Workspace Design.

[40] Helander M.G., Willén B.Å., Karwowski W., Marras W.S. (1999). Design for Human Assembly (DHA). The Occupational Ergonomics Handbook.

[41] Kroemer K.H.E., Kroemer A.D. (2001). Office Ergonomics.

[42] Kroemer K.H.E., Kroemer H.B., Kroemer-Elbert K.E. (2000). Ergonomics: How to Design for Ease and Efficiency.

[43] Sanders M.S., McCormick E.J. (1993). Human Factors in Engineering and Design.

[44] Wickens C.D., Lee J., Gordon-Becker S., Liu Y. (2014). An Introduction to Human Factors Engineering.

[45] Kroemer K.H.E. (1999). Assessment of Human Muscle Strength for Engineering Purposes: A Review of the Basics. Ergonomics.

[46] Noyes J., Garland K., Bruneasu D., Sandorn C., Harvey R.S. (2004). Humans: Skills, Capabilities and Limitations. Human Factors for Engineers.

[47] Noyes J. (2002). Psychology at Work. Designing for Humans.

[48] Wilson J.R., Sharples S. (2015). Evaluation of Human Work.

[49] Lopez K.D., Gerling G.J., Cary M.P., Kanak M.F. (2010). Cognitive Work Analysis to Evaluate the Problem of Patient Falls in an Inpatient Setting. JAMIA.

[50] Hollnagel E., Braithwaite J., Wears R.L. (2013). Resilient Health Care.

[51] Colman N., Dalpiaz A., Hebbar K.B. (2020). Simulation Enhances Safety Evaluation in the Design of New Healthcare Facilities. Curr. Treat. Options Peds..

[52] Jurewicz K.A., Neyens D.M., Catchpole K., Joseph A., Reeves S.T., Abernathy J.H. (2021). Observational Study of Anaesthesia Workflow to Evaluate Physical Workspace Design and Layout. Br. J. Anaesth..

[53] Bayramzadeh S., Aghaei P. (2021). Technology Integration in Complex Healthcare Environments: A Systematic Literature Review. Appl. Ergon..

[54] RIPCHD.OR Study Group (2016). Realizing Improved Patient Care through Human-Centered Design in the OR.

[55] Taylor E. (2017). The Healthcare Workplace: More Than a New ‘Old’ Hospital. J. Inter. Des..

[56] Piatkowksi M., Taylor E., Wong B., Taylor D., Foreman K.B., Merryweather A. (2021). Designing a Patient Room as a Fall Protection Strategy: The Perspectives of Architects. Int. J. Environ. Res. Public Health.

